# The variation profile of associated microbiota in juvenile whelk *Hemifusus tuba* (Gmelin, 1791) in dietary transition

**DOI:** 10.3389/fmicb.2026.1843060

**Published:** 2026-05-28

**Authors:** Yongli Wu, Zijian Lu, Shaobo Ma, Huixian Zhang, Wenwen Zhang, Xilan Ma, Kai Yuan, Jiangyong Wang

**Affiliations:** 1 College of Life Science, Huizhou University, Huizhou, China; 2 State Key Laboratory for Marine Environmental Science, College of Ocean and Earth Sciences, Xiamen University, Xiamen, China; 3 CAS Key Laboratory of Tropical Marine Bio-resources and Ecology, South China Sea Institute of Oceanology, Chinese Academy of Sciences, Guangzhou, China; 4 South China Sea Ecology Center, Ministry of Natural Resources, Guangzhou, China

**Keywords:** associated microbiota, core microbiome, dietary transition, functional prediction, Hemifusus tuba

## Abstract

*Hemifusus tuba* (Gmelin, 1791) is a commercial marine gastropod that undergoes an ontogenetic dietary transition from egg-capsule nutrition (lecithotrophy) to herbivory and finally to lifelong carnivory. How associated microbiota respond to this feeding habit transition remained largely unexplored. Here, we characterized taxonomic composition, diversity and functional prediction of associated microbiota in *H. tuba* across three dietary stages (CK, egg-capsule nutrition or lecithotrophic; He, herbivory; Ca, carnivory) using 16S rRNA amplicon sequencing. A total of 457,412 high-quality reads from 14 libraries were clustered into 12,091 OTUs, identifying 811 genera within 38 phyla. And five dominant phyla (*Proteobacteria*, *Bacteroidota*, *Firmicutes*, *Actinobacteriota*, and *Planctomycetota*) accounted for 99% of total abundance and formed the core microbiota. Alpha diversity increased from CK to He and peaked in Ca, whereas beta diversity analyses consistently separated Ca from CK and He, indicating microbiota restructuring following the transition to carnivory. During the feeding habit transition, *Proteobacteria* and *Planctomycetota* increased, while *Bacteroidota*, *Firmicutes*, and *Actinobacteriota* declined. LEfSe identified *Hyphomicrobiales*/*Bradyrhizobiaceae*, *Burkholderiales*/*Betaproteobacteria*, and *Rhodobacterales*/*Roseobacteraceae* as biomarkers for CK, He, and Ca, respectively. PICRUSt2 functional predictions indicated that CK enriched for tryptophan and butyrate metabolism and fatty acid degradation, whereas Ca appeared to be enriched for methane and pyruvate metabolism and the TCA cycle. These diet-associated microbiome shifts might facilitate nutrient utilization and energy metabolism during feeding habit transition, and provided microbial candidates for feed optimization in *H. tuba* domestication.

## Introduction

Animal-associated microbiota are complex communities of bacteria, archaea, fungi, and viruses that colonize host tissues and contribute to nutrition, metabolism, development, and health (Wei et al., 2018). Among the factors shaping these microbial communities, diet is one of the most important drivers of microbiota structure and function (Makwarela et al., 2025). In mollusks, increasing evidence indicated that dietary variation could alter microbial diversity, community composition, and predicted metabolic potential. For example, the freshwater snail *Planorbella trivolvis* harbored diet-dependent associated microbiota, with high-fiber herbivorous diets supporting diverse communities enriched in cellulose- and lignocellulose-degrading taxa, while low-fiber diets reduced diversity and increased *Proteobacteria* dominance (Hu et al., 2021). And the diet of the invasive apple snail *Pomacea canaliculata* shifted with seasons and habitats, leading to compositional changes in its associated microbial communities that helped the host adapt to diverse environments and enhance its invasive success (He et al., 2025; Yao et al., 2025). In marine gastropod, *Haliotis discus hannai* exhibited changes in intestinal microbiota and improved intestinal health when fed diets supplemented with different levels of bile acids (Chen et al., 2023). Collectively, host-related dietary factors not only shaped microbial diversity and community composition but also influenced host physiology, immune responses, and resilience to environmental stressors (Makwarela et al., 2025). However, most studies have focused on adult or juvenile animals under relatively stable feeding conditions, whereas microbiota dynamics during ontogenetic dietary transition remain poorly understood.

*Hemifusus tuba* (Gmelin, 1791), commonly known as the tuba false fusus, is a large predatory marine gastropod of economic and aquaculture interest (Morton, 1985; Zhou et al., 2018). Female *H. tuba* lay egg capsules attached to hard substrates and early embryonic development proceeds entirely within the egg capsules, where embryos depend on intracapsular nutrients, including intracapsular fluid and nurse eggs, to support embryogenesis and larval development (Han et al., 2022). After hatching, juveniles undergo a clear ontogenetic dietary transition: newly hatched individuals first shift to herbivory, feeding on benthic diatoms, and then subsequently transition to carnivory, feeding on fresh bivalve tissue (Lu et al., 2009). Thus, juvenile *H. tuba* experiences a distinct sequence of nutritional stages, from capsule-based endogenous nutrition (lecithotrophy) to herbivory and finally to persistent carnivory.

This ontogenetic dietary shift in *H. tuba* provides a model for investigating microbiota dynamics across developmental feeding stages. However, the dynamics of the associated microbiota in *H. tuba* during ontogenetic transition remain poorly understood. Particularly, it is unclear how the associated microbial community changes across the shift from lecithotrophy to herbivory and subsequently to carnivory, whether specific associated microbial taxa are associated with specific feeding stages, and how predicted microbial functions and community assembly processes shift during transition. This knowledge gap limits our understanding of the relationship between dietary transition and associated microbiota in marine gastropods and restricts the potential application of microbiome-based strategies in *H. tuba* aquaculture.

In this study, we investigated the associated microbiota dynamics in *H. tuba* across its feeding habit transition. The specific objectives were to: (1) characterize changes in associated microbiota diversity and community composition associated with dietary transition; (2) identify dominant and key bacteria taxa with different diets; and (3) investigate the changes in microbiome assembly processes and predicted functional phenotypes during the feeding habit transition from lecithotrophic nutrition to herbivory and subsequently to carnivory.

## Materials and methods

### Rearing conditions and sampling

Broodstock of *H. tuba* were maintained in a fish farm in Jieyang, China. After laying of the egg capsules by the whelk, strings of egg capsules were removed carefully to avoid damage and then raised in the marine aquaculture system of the Huizhou University (Huizhou, China). Based on these literatures (Morton, 1986; Han et al., 2022), the culture conditions were kept at a temperature of 23 ± 0.5 °C and a salinity of 27% ± 1‰ to provide relatively stable and suitable rearing conditions for juvenile *H. tuba* during the experiment, thereby minimizing environmental stress and avoiding confounding effects on microbiota variation. The status of the eggs/developing embryo inside each capsule was observed and recorded every day. After about 45 days of incubation, the hatchlings inside the egg capsules almost developed with complete organ but still used intracapsular fluid nutritional composition as energy sources and were defined as the lecithotrophic stage (CK group). The juvenile shed from the egg capsules after about 47 ± 3 days of incubation, then were subsequently fed with *Chlorella* as food for 3 days (herbivorous stage, He group). Subsequently, the juveniles were fed fresh clam meat for 5 days and were defined as the carnivorous group (Ca group). Dead individuals in Ca group were removed promptly to avoid cannibalism. Five juvenile individuals from each group (CK, He, and Ca) were randomly selected to measure total shell length (SL) and shell width (SW) using a vernier caliper. The measured juvenile had a mean SL of 12.72 ± 0.51 mm and a mean SW of 7.40 ± 0.60 mm.

Because juvenile *H. tuba* were too small to dissect the gut or other regions of the digestive system, the associated microbiota analyzed in this study was obtained from the whole soft body after shell removal rather than from an isolated gut sample. For sampling, five individual snails were pooled as one biological replicate, and five replicates were prepared for each group. In the CK group, egg capsules were opened using sterilized scissors, and five juveniles were collected from egg capsules within the same egg string. In the He and Ca groups, five juveniles were randomly collected from the corresponding rearing tanks of that stage. All juveniles were suspended 3 times in sterile 0.01 M phosphate buffered saline (PBS) at pH 7.2. The shells were removed, and the soft bodies were washed 3 times with sterile 0.01 M PBS. Five individual soft bodies from the same feeding group were pooled into a single DNase/RNase-free microcentrifuge tube.

### DNA extraction and PCR amplification

The pooled soft bodies from each tube were homogenized, and microbial DNA was extracted using the E. Z. N. A.® Soil DNA Kit (Omega Bio-tek, Norcross, GA, USA) following the manufacturer’s protocols. The V1-V9 region of the bacterial 16S ribosomal RNA gene was amplified by PCR with 27 cycles at 95 °C for 30 s, 55 °C for 30 s, and 72 °C for 60 s, followed by a final extension at 72 °C for 5 min. The PCR reaction mixture (20 μL) consisted of 4 μL of 5 × FastPfu Buffer, 2 μL of 2.5 mM dNTPs, 0.8 μL of each primer (5 μM), 0.4 μL of FastPfu Polymerase, and 10 ng of template DNA. The full-length bacterial 16S rRNA gene was amplified using the universal primers 27F (5’-AGRGTTYGATYMTGGCTCAG-3′) and 1492R (5’-RGYTACCTTGTTACGACTT-3′), which are widely used for bacterial 16S rRNA gene amplification (Weisburg et al., 1991). Amplicons were extracted from 2% agarose gels and purified using the AxyPrep DNA Gel Extraction Kit (Axygen Biosciences, Union City, CA, USA) as per the manufacturer’s instructions.

### Library construction, sequencing and processing of sequencing data

SMRTbell libraries were prepared from the amplified DNA by blunt-ligation according to the manufacturer’s instructions (Pacific Biosciences). The purified SMRTbell libraries from the Zymo and HMP mock communities were sequenced on dedicated PacBio Sequel II 8 M cells using the Sequencing Kit 2.0 chemistry. The pooled and barcoded samples were sequenced on a single PacBio Sequel II cell. All sequencing was conducted by Shanghai Biozeron Biotechnology Co. Ltd. (Shanghai, China).

PacBio raw reads were processed using the SMRT Link Analysis software version 9.0 to generate demultiplexed circular consensus sequence reads with the following parameters: a minimum of three passes and a minimum predicted accuracy of 0.99. Raw reads were further processed through SMRT Portal to filter sequences for length (≥2,500 bp) and quality. Sequences were additionally filtered by removing barcodes, primer sequences, chimeras, and sequences containing 10 or more consecutive identical bases.

Operational taxonomic units (OTUs) were clustered using UPARSE (version 7.1) (Edgar, 2013) at a 98.65% sequence similarity threshold, and chimeric sequences were identified and removed using UCHIME (Edgar et al., 2011). We used an OTU-based workflow rather than an amplicon sequence variant (ASV)-based denoising workflow. This threshold was selected because the present study was based on full-length 16S rRNA gene sequences generated by third-generation sequencing, and a 98.65% similarity cutoff has been used to approximate species-level resolution for full-length 16S rRNA sequences. Representative sequences of each OTU were taxonomically assigned using the RDP Classifier (Wang et al., 2007) against the SILVA SSU132 16S rRNA database (Quast et al., 2013) with a confidence threshold of 70%.

### Bioinformatics and statistical analyses

Alpha diversity was evaluated using richness indices, including Observed OTUs, Chao1, and ACE; diversity and evenness indices, including Shannon, Simpson, and Pielou’s J; and phylogenetic diversity, including Faith’s PD. Considering the small and unbalanced sample size among groups, differences in alpha diversity indices among the three dietary stages (CK, He, and Ca) were assessed using the non-parametric Kruskal–Wallis test. When the overall test was significant, Dunn’s *post hoc* test with Benjamini–Hochberg correction was used for pairwise comparisons. Beta diversity was computed based on Bray–Curtis dissimilarity and both unweighted and weighted UniFrac distances and visualized by principal coordinates analysis (PCoA). Differences in community structure among stages were tested using permutational multivariate analysis of variance (PERMANOVA) (Anderson, 2001) with 999 permutations. Stage-associated taxa were further identified using Tukey’s HSD test on the top-ranked taxa at multiple taxonomic levels, and biomarker taxa were determined by linear discriminant analysis effect size (LEfSe) with an alpha value of 0.05 and an LDA score threshold of 2.0 (Segata et al., 2011).

Functional profiles were inferred from 16S rRNA data using PICRUSt2 (Douglas et al., 2020) to predict KEGG Orthology abundances and collapse them into KEGG pathway-level profiles. Differentially abundant pathways among stages were identified using appropriate multiple-group statistical tests, and *p* values were adjusted for multiple comparisons where applicable. Unless otherwise stated, statistical significance was set at *p* < 0.05. All analyses and visualizations were conducted in R (version 4.5.2) with packages commonly used for microbial community ecology.

## Results

### Characteristics of sequencing data

Five biological replicates were initially prepared for each group. During downstream analysis, one sample from the He group was identified as an outlier based on PCA and was therefore excluded from subsequent analyses. As a result, four samples were retained for the He group. Sequencing of the 14 qualifying samples produced 457,412 high-quality reads, with an average read length of 1435.10 base pairs. These reads were then clustered, yielding 12,091 OTUs, which are slated for further bioinformatics analysis.

### The dominant bacterial taxa of microbiota in three diet-stage groups

**Figure 1:**
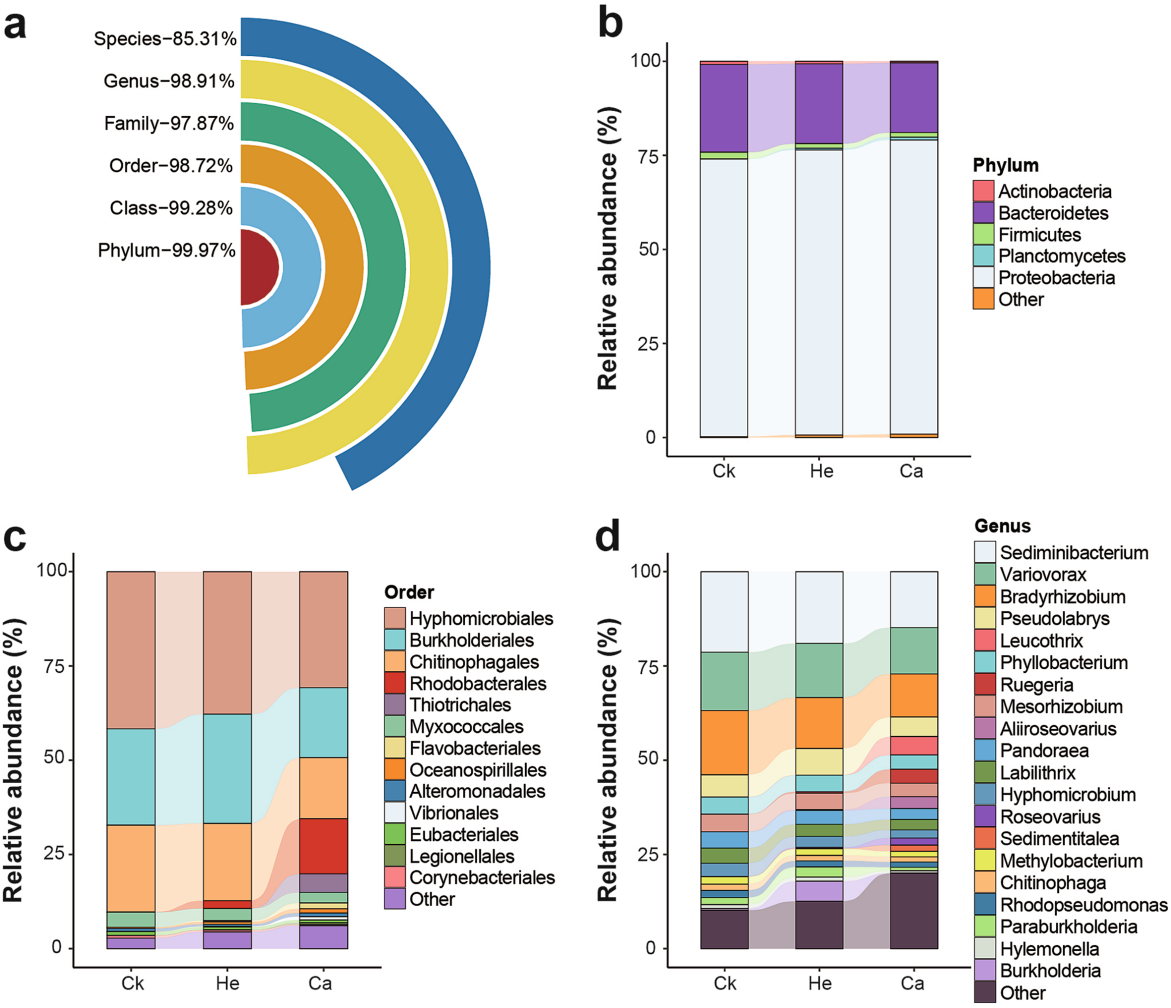
Annotation proportions and inter-group community composition. **(a)** Proportions of annotated OTUs across various taxonomic ranks. **(b–d)** Comparative structural analysis of microbial communities among CK, He, and Ca groups at the phylum, order, and genus levels, respectively.

To investigate the differences of *H. tuba* microbiota in different diet-stage, species composition was analyzed. Species annotations from 16S rRNA sequencing showed that most OTUs could be taxonomically classifiable (Figure 1a). More than 97.87% of the OTUs could be categorized at the phylum, class, order, family, and genus levels; however, the percentage of species-level annotation dropped to 85.31%.

A total of 38 phyla were annotated, of which the top five identifiable dominant phylum including *Proteobacteria*, *Bacteroidetes*, *Firmicutes*, *Actinobacteria* and *Planctomycetes* accounted for 99.0% of the total abundance ratio (Figure 1b). These formed the core microbiota of *H. tuba*. As the feeding habits of *H. tuba* transitioned from lecithotrophy (CK) to herbivorous (He) and finally to carnivorous (Ca), there was a gradual increase in the proportion of *Proteobacteri*a and *Planctomycetes*, while the proportions of *Bacteroidetes*, *Firmicutes*, and *Actinobacteria* decreased.

A total of 128 orders were annotated. The dominant identifiable bacteria were *Hyphomicrobiales*, *Burkholderiales*, *Chitinophagales* and *Rhodobacterales* (Figure 1c). Together, these four taxa accounted for 90.3%, 89.3%, and 80.2% of the CK, He, and Ca groups, respectively. *Rhodobacterales* showed a clear increase in the Ca group, accounting for 14.62% of the community, compared with only 0.04% in CK and 2.05% in He.

At the genus level, there were 811 taxa annotated. *Sediminibacterium*, *Bradyrhizobium*, Var*iovorax*, *Pseudolabrys*, and *Mesorhizobium* were noteworthy taxa across three dietary stages (Figure 1d). Notably, some bacterial genera exhibited distinct proportions among the groups. For instance, *Burkholderia* constituted 5.4% of the He group, while its presence was only 0.51% and 0.06% in the CK and Ca groups, respectively. *Aliiroseo*var*ius* represented 3.2% of the total abundance in the Ca group, contrasting sharply with its 0.005% and 0.19% in the CK and He groups. *Ruegeria* displayed a similar pattern to *Aliiroseovarius*, demonstrating a higher abundance exclusively in the Ca group.

### Alpha and beta diversity analysis of microbial communities in *Hemifusus tuba* across three dietary stages

The rarefaction and Shannon-Wiener curves indicated that as sequencing data volume increased (Supplemental Figure S1), both curves approached a plateau, suggesting that the sequencing depth was ample to represent most of the microbial information in the samples.

In the analysis of associated microbiota alpha diversity across different feeding stages in *H. tuba*, significant overall variations were observed across all seven indices based on the Kruskal–Wallis test. The richness of the microbial community, as assessed by the Observed species, Chao1, and ACE indices, was generally highest in the Ca group and lowest in the CK group, with the He group showing intermediate values (Table 1). Dunn’s *post hoc* test with Benjamini–Hochberg correction further showed that the Ca group had significantly higher Observed species, Chao1, and ACE values than the CK group, while differences involving the He group were less consistent. A similar pattern was observed for diversity indices. The Shannon, Simpson, Pielou’s J, and Faith’s PD indices were significantly higher in the Ca group than in the CK group, whereas the He group generally showed intermediate values and was not consistently different from either CK or Ca after multiple-testing correction. Collectively, these results suggest an overall increase in microbiota richness and diversity during the dietary transition of *H. tuba* from lecithotrophic nutrition to herbivory and subsequently to carnivory, although the pairwise differences did not support a strictly stepwise significant increase across all three stages for every index.

**Table 1:** Richness and diversity indices of the 16S rRNA gene from *Hemifusus tuba* across three dietary patterns (mean ± standard deviation).

Diet stages	Richness index			Diversity index			
	Observed species	Chao1	ACE	Shannon	Simpson	Pielou’s *J*	Faith’s PD
CK	1069.40 ± 123.48^a^	1465.32 ± 304.85^a^	1652.37 ± 356.35^a^	4.04 ± 0.10^a^	0.96 ± 0.001^a^	0.58 ± 0.01^a^	41.16 ± 5.56^a^
He	1404.25 ± 46.03^ab^	1534.48 ± 64.39^ab^	1700.93 ± 94.79^a^	4.35 ± 0.14^ab^	0.97 ± 0.004^ab^	0.6 ± 0.02^a,b^	55.54 ± 4.43^a,b^
Ca	1733.00 ± 110.58^b^	1960.18 ± 134.21^b^	2195.68 ± 159.35^b^	4.9 ± 0.16^b^	0.98 ± 0.003^b^	0.66 ± 0.02^b^	65.74 ± 6.15^b^

Values are shown as mean ± SD. Different superscript letters in the same column indicate significant differences among dietary stages based on Dunn’s post hoc test with Benjamini–Hochberg correction after a significant Kruskal–Wallis test (*p* < 0.05). Different superscript letters (a, b) indicate statistically significant differences between groups.

**Figure 2:**
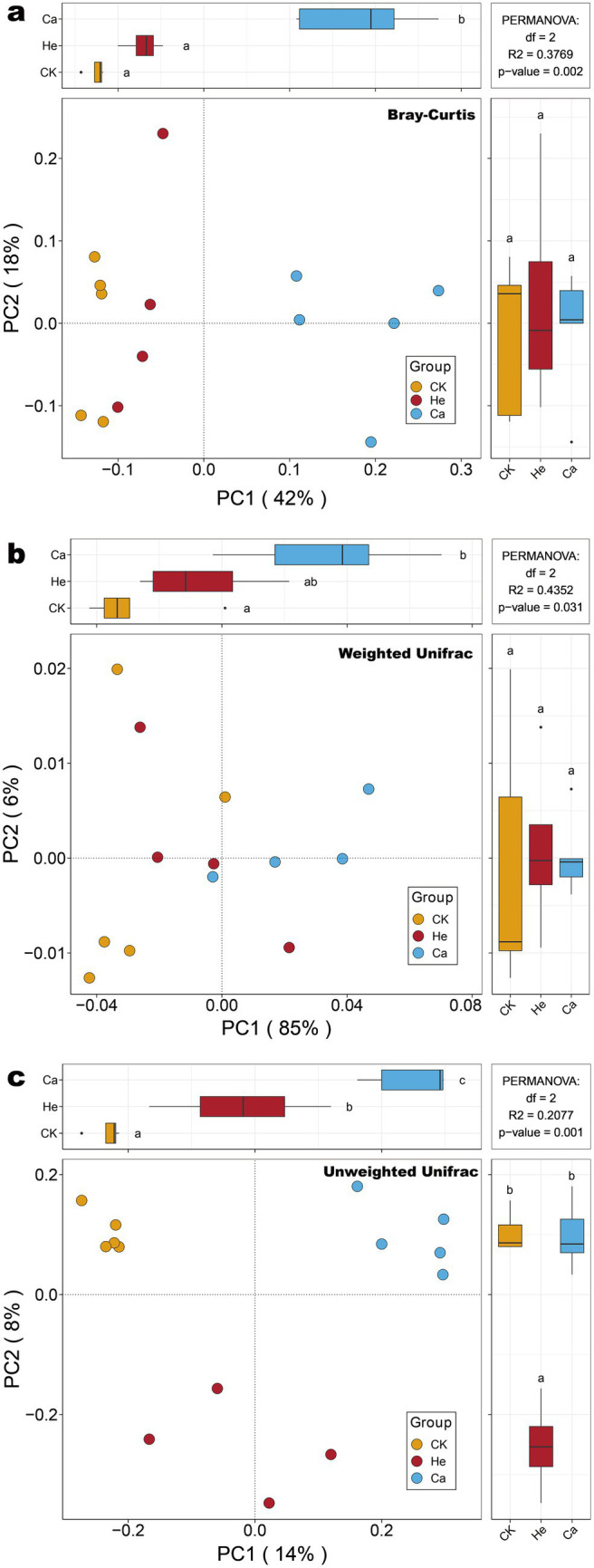
PCoA-based beta diversity analysis. **(a–c)** Depict the outcomes using Bray-Curtis, weighted Unifrac, and unweighted Unifrac distances, respectively. Distinct colors denote different diet-stage groups. Box plots at the top and right corners display beta diversity distances across groups on PC1 and PC2, with different letters signifying significant differences (*p* < 0.05, Tukey’s HSD test). The top right corner presents PERMANOVA (adonis) test outcomes.

In the beta diversity analysis of 16S rRNA gene sequencing data using principal coordinate analysis (PCoA), subtle variations were noted across three distinct indices (Figure 2). The Bray–Curtis dissimilarity metric revealed that Group Ca was distinctly separated from Groups He and CK along the first principal coordinate axis, with no significant differences observed among the groups along the second axis (Figure 2a). The Unweighted Unifrac distance metric demonstrated significant inter-group variation across both the first and second principal coordinate axes, indicating effective discrimination among the three groups (Figure 2c). In contrast, the Weighted Unifrac distance metric identified significant differences exclusively between Group Ca and Group CK along the first principal coordinate axis (Figure 2b). Integrating the results from these three indices, we found that the microbial community composition of Group Ca is markedly distinct from the other two groups. These findings suggested a substantial shift in the associated microbiome of *H. tuba* following its dietary transition towards carnivory.

### Difference and similarity of associated microbiota among three dietary stages

To investigate the variations in associated microbiota across three diet-stage groups, we conducted Tukey’s HSD test to analyze the differences among the top 10 taxa in terms of relative abundance identified at various taxonomic levels. At the phylum level, we noted notable variations in the relative abundance of *Acidobacteria*, *Chloroflexi*, *Planctomycetes*, and *Verrucomicrobia* (Supplemental Figure S2a). In particular, the CK group had a significantly lower proportion of *Acidobacteria* compared to the other groups. Furthermore, the CK group also showed significantly reduced levels of *Chloroflexi*, *Planctomycetes*, and *Verrucomicrobia* compared to the Ca group. At the order level, differences in relative abundance were significant for *Burkholderiales*, *Chitinophagales*, *Flavobacteriales*, *Hyphomicrobiales*, *Rhodobacterales*, and *Thiotrichales* (Supplemental Figure S2b). Specifically, the abundance of *Burkholderiales* was significantly higher in the He group than in the Ca group. The CK group had a significantly higher abundance of *Chitinophagales* and *Hyphomicrobiales* than the Ca group. Conversely, the Ca group had a significantly higher abundance of *Flavobacteriales*, *Rhodobacterales*, and *Thiotrichales* compared to both the He and CK groups. At the genus level, considerable shifts in relative abundance were detected for *Bradyrhizobium*, *Hyphomicrobium*, and *Sediminibacterium* (Supplemental Figure S2c). Notably, all three genera were more abundant in the CK group.

**Figure 3:**
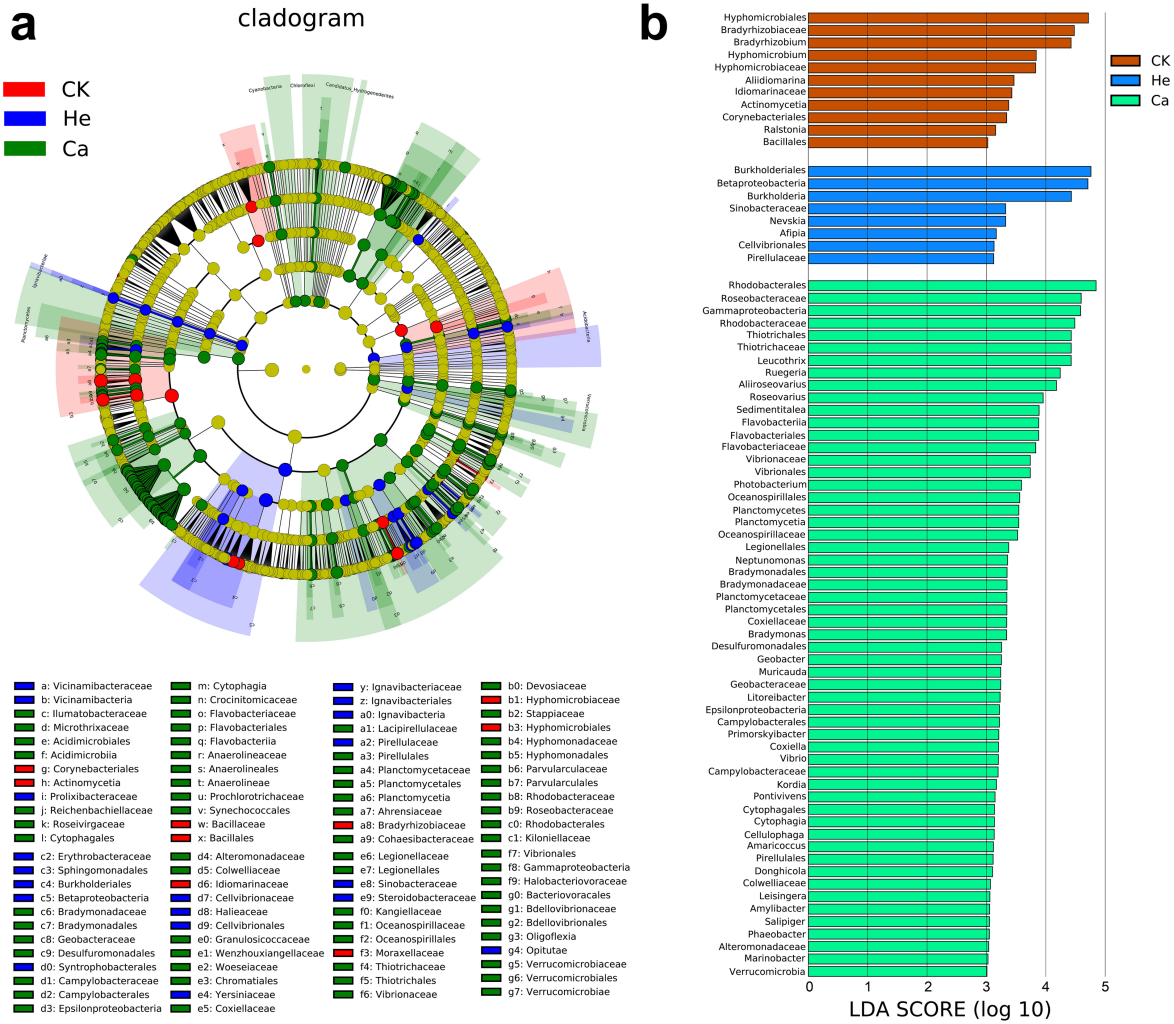
LEfSe analysis reveals the most significantly varying taxa across the three dietary habit stages. **(a)** The taxonomic cladogram. This diagram illustrates the taxonomic distribution from phylum to genus/species. Circle size corresponds to relative abundance, with larger circles indicating higher prevalence. Non-significant species are marked in yellow, while significant biomarkers are color-coded by group: CK (red), He (blue), and Ca (green). Species names denoted by letters are detailed in the legend below the figure. **(b)** LDA scores for enriched taxa. Taxa with significant enrichment in CK (red), He (blue), and Ca (green) groups are presented, filtered to include only those surpassing an LDA score threshold of 3.

Utilizing LEfSe, we uncovered 120 key taxonomic variations between the three diet-stage groups, highlighting several phylotypes as microbiological markers at different phylogenetic levels (Figure 3a). These markers included *Hyphomicrobiales* and *Bradyrhizobiaceae*, which were distinctive of the CK group when compared to the other two groups. Similarly, *Burkholderiales* and *Betaproteobacteria* were found to be indicative of the He group, setting it apart from the others. Lastly, *Rhodobacterales* and *Roseobacteraceae* were identified as markers that could differentiate the Ca group from the other two groups (Figure 3b).

### Microbiota function

**Figure 4:**
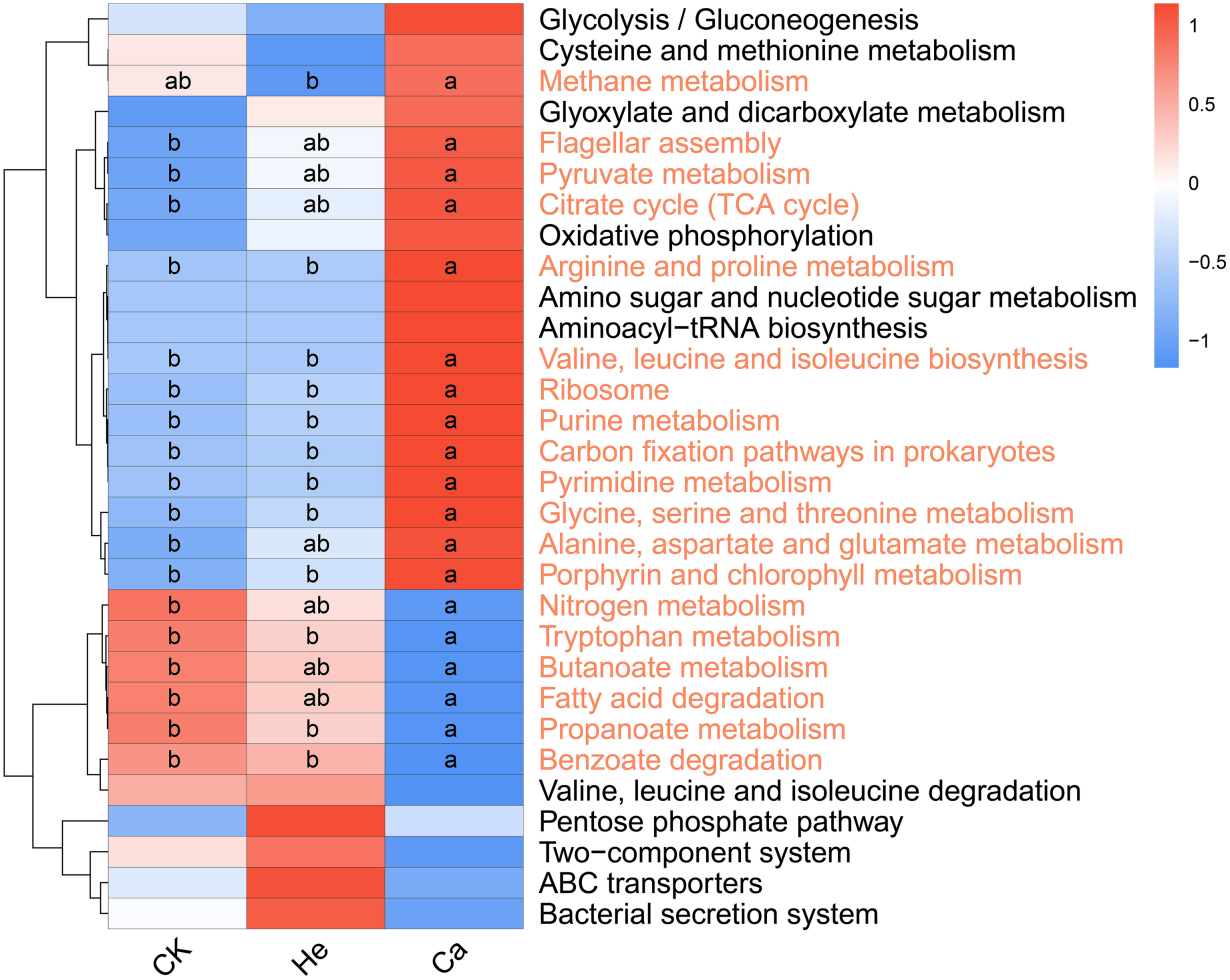
Heatmap of dominant microbial functional pathways. Only the top 30 KEGG pathways, ranked by microbial functional abundance, across the three diet-stage groups are displayed. Groups labeled with different letters indicate statistically significant differences in pathway abundance (*p* > 0.05, Tukey’s HSD test).

PICRUSt2 was employed to predict the functional profiles of the microbiota in the *H. tuba*. We identified 251 KEGG pathways that displayed similar gene functions but showed variations in abundance across the three diet groups. The 30 most abundant pathways mainly represented core metabolic functions, including carbohydrate, lipid, and amino acid metabolism, energy production, nucleotide metabolism, specialized metabolic processes, and cellular transport and signaling (Figure 4). Additionally, 19 pathways exhibited statistically significant differences (*p* < 0.05) in relative abundance among the diet groups.

Upon further comparison, PICRUSt2-based prediction suggested that the CK group had higher predicted abundances of pathways related to tryptophan metabolism, butanoate metabolism, and fatty acid degradation. Conversely, The Ca group indicated higher predicted abundances of pathways related to methane metabolism, pyruvate metabolism, and the citrate cycle (TCA cycle). The He group appeared to be more enriched in the pentose phosphate pathway, two-component system, ABC transporters, and bacterial secretion system. However, statistical analysis indicated no significant differences in these four pathways between the He group and the other two groups.

## Discussion

Our study provided a comprehensive characterization of microbial succession across three dietary stages in *H. tuba*, revealing coordinated taxonomic and functional restructuring accompanying the dietary transition from lecithotrophy to herbivory and ultimately carnivory. Our findings demonstrated that dietary shift was associated with progressive increases in microbial diversity, marked compositional divergence in the carnivorous stage, and functional reconfiguration of metabolic pathways, highlighting a tight coupling between host nutritional strategy and microbiome assembly during ontogeny.

### Core microbiota stability and diet-associated restructuring

Despite diet transitions, the associated microbiota of *H. tuba* was dominated by a relatively stable core framework at the phylum level, primarily composed of Proteobacteria, Bacteroidota, Firmicutes, Actinobacteriota, and Planctomycetota (Figure 1b), suggesting the presence of a conserved microbiome throughout development. For instance, Proteobacteria, Bacteroidota and Firmicutes identified in some snails are considered to constitute the “core” intestinal microbiota, which their implicated functions in nutrient assimilation, metabolic processing (Yang et al., 2022; Chen et al., 2023; Makwarela et al., 2025). However, the relative abundance changed in dietary transitions. Proteobacteria and Planctomycetota increased from the lecithotrophy (CK) to herbivorous (He) and finally to carnivorous, whereas Bacteroidota, Firmicutes, and Actinobacteriota declined (Figure 1b). This was consistent with previous studies. Research on the associated microbiota of *Planorbella trivolvis* fed different diets found that, compared to the low-fiber non-herbivorous diet group, the high-fiber herbivorous diet group had a lower abundance of Proteobacteria but a higher abundance of Bacteroidota (Hu et al., 2021). Therefore, the observed microbial communities restructuring occurs on dietary transitions in *H. tuba*.

### Diet-specific taxa and functional reconfiguration of the microbiome

LEfSe analysis combined with Tukey’s HSD test identified three dietary-specific microbial markers, key indicators capable of distinguishing between different dietary transitions (Figure 3a). In the lecithotrophic individuals, our results showed that *Hyphomicrobiales* and its family *Bradyrhizobiaceae* were significantly observed. At the genus level, *Bradyrhizobium* and *Sediminibacterium* were dominant (Figure 3a). The enrichment of these microbial taxa during the lecithotrophic stage, a period characterized by larval development within enclosed egg capsules and the absence of exogenous nutrient intake, indicated that microbial colonization might be already established before exogenous feeding. Such early colonization might arise through multiple routes. One possible explanation was vertical transmission, which has been reported in other animals as a mechanism by which microorganisms become associated with the host during oogenesis or vitellogenesis and persist through embryonic and larval development (Fiedler et al., 2023; Lin et al., 2023; Scanes et al., 2023; Chervochkina et al., 2025), although this possibility was not directly tested in the present study. The predicted functional profile of the CK microbiota further suggested the possibility of early host–microbe metabolic interactions. PICRUSt2 analysis indicated enrichment of multiple metabolic pathways, including fatty acid degradation, butanoate metabolism, propanoate metabolism, nitrogen metabolism, tryptophan metabolism, and benzoate degradation (Figure 4). Collectively, these pathways might reflect enhanced microbial potential for nutrient turnover and energy conversion during the lecithotrophic stage. Importantly, these bacteria might contribute to the degradation of yolk-derived lipoproteins and proteins, thereby supplying macromolecules and energy substrates required for the development of *H. tuba*. Evidence from gnotobiotic fish models indicated that microbiota could enhance fatty acid uptake and lipid deposition, supporting a potential role of associated microbes in yolk-derived nutrient utilization during early development (Semova et al., 2012). This interpretation is consistent with the PICRUSt2 predictions (Figure 4), which indicated the enrichment of fatty acid degradation during the lecithotrophic stage. Furthermore, PICRUSt2 predictions revealed an enrichment of butyrate metabolism pathways (Figure 4), which was consistent with the known role of associated microbiota in short-chain fatty acid metabolism, including butyrate production, in Atlantic salmon (Vargas et al., 2023). Likewise, the enrichment of propanoate metabolism suggested potential shifts in short-chain fatty acid-related carbon metabolism, which could contribute to microbial energy conversion and host–microbe metabolic exchange during the nutritionally enclosed lecithotrophic stage (Den Besten et al., 2013). In addition, the enrichment of nitrogen metabolism might indicate an association with microbial transformation of nitrogen-containing substrates and amino acid turnover (Glaze et al., 2022), thereby contributing to nutrient recycling and host–microbe metabolic exchange during the lecithotrophic stage and the herbivorous stage. The predicted enrichment of tryptophan metabolism was also remarkable, as microbial tryptophan metabolism has been linked to host metabolic and intestinal functions like in pearl gentian groupers (Zhang et al., 2022). Benzoate degradation might further suggest an enhanced microbial potential for degrading aromatic organic compounds, indicating a broader substrate utilization capacity in the CK-associated microbiota, as also reported in *Sthenoteuthis oualaniensis* (Hu et al., 2022). Together, these taxonomic and functional patterns suggested that host–microbe metabolic interactions could already be established in *H. tuba* during the lecithotrophic stage. However, because the PICRUSt2 functional predictions were inferred solely from 16S rRNA gene data and lacked experimental validation, they might not accurately reflect actual metabolic activity.

In the herbivorous individuals, the phylum *Burkholderiales* and *Betaproteobacteria* were significantly enriched (Figure 3b), the taxa commonly linked to plant-associated environments and involved in nitrogen metabolism and membrane transport (Pérez-Pantoja et al., 2012). At the genus level, *Burkholderia* accounted for 5.4% of the microbial community in the herbivorous individuals, compared with less than 0.6% in both the lecithotrophic and carnivorous individuals (Figure 1d). Members of *Burkholderia* are recognized for its remarkable metabolic versatility, with some strains capable of degrading cellulose derivatives and other plant-derived polysaccharides (Diyapoglu et al., 2025). As observed in other mollusks, the abundance of *Burkholderia* and some microbes shifted in response to dietary changes (Shi et al., 2020; Yang et al., 2022). The increased abundance of *Burkholderia* in the herbivorous individuals therefore suggested a diet-mediated restructuring of the microbiota. Specifically, *Burkholderia’s* enrichment might reflect a microbial adaptation of *H. tuba* to benthic diatoms diets (Lu et al., 2009) rich in complex carbohydrates, potentially facilitating the degradation and assimilation of plant polysaccharides (Diyapoglu et al., 2025). These findings might support the hypothesis that *H. tuba* during the herbivorous phase relies on associated microorganisms with polysaccharide-degrading capacities to compensate for the host’s lower nutrient intakes through microbial action to maximize energy utilization. The predicted enrichment of the pentose phosphate pathway in the He group (Figure 4) further supports this interpretation. The pentose phosphate pathway is a central carbohydrate metabolic pathway that could be for sugar interconversion (Kanehisa et al., 2025). Its enrichment might therefore indicate an increased microbial capacity for processing algal-derived carbohydrates after the transition to exogenous feeding. This pattern suggested that the He-associated microbiota might contribute not only to the utilization of plant- and algal-derived carbon sources, but also to microbial biosynthesis and host–microbe metabolic exchange during the herbivorous stage in *H. tuba*, as supported by studies on marine fish associated microbiomes (Egerton et al., 2018; Herrera et al., 2022).

At the order level, our results revealed that as *H. tuba* transitioned to a carnivorous diet, the abundance of *Rhodobacterales* increased significantly (Figure 1c), an order which was also commonly found in other marine carnivores (Sullam et al., 2012). Consistent with this result, LEfSe analysis identified *Rhodobacterales* and the family *Roseobacteraceae* as microbial markers of the carnivorous individuals (Figure 3a). *Rhodobacterales* have been contributed to growth promotion in marine invertebrates, potentially due to their abundance of *Rhodobacteraceae* and possession of polyhydroxybutyrate metabolism genes (Yamazaki et al., 2016). Moreover, *Rhodobacteraceae* played an important role in food habit transition in carnivorous gastropods such as *Rapana venosa* (Yang et al., 2022), highlighting their relevance during dietary shifts. Many members of the *Rhodobacterales* (including *Aliiroseovarius* and *Ruegeria*) were metabolically versatile aerobic heterotrophs with strong central carbon metabolic potential. Genomic analyses of marine *Rhodobacterales* have shown broadly conserved complete tricarboxylic acid (TCA) cycle components and diverse respiratory cytochrome systems, consistent with efficient carbon oxidation and energy generation (Wu et al., 2025). Consistent with *Rhodobacterales’* metabolic background, PICRUSt2 predictions might reveal significant enrichment of pathways potentially associated with the TCA cycle, pyruvate metabolism, and methane metabolism in carnivorous individuals (Figure 4). These predicted patterns might be compatible with potential changes in microbial carbon flux and energy-related functions under a diet enriched in protein- and lipid-rich animal prey, but they might have required validation by metagenomic, metatranscriptomic, or metabolomic approaches. Furthermore, the increased relative abundances of *Aliiroseovarius* and *Ruegeria* suggested that carnivorous feeding might impose stronger selective pressures favoring microbial taxa with high metabolic efficiency and oxidative metabolic capacity. This interpretation was also consistent with diet–microbiome studies showing that animal-protein/fat-rich feeding regimes could enrich microbial functions associated with central carbon metabolism, fatty acid oxidation, and energy harvesting, whereas herbivorous diets are more often associated with fermentative/pyruvate-centered carbohydrate metabolism (Wu et al., 2025). And these observations suggested that the carnivorous diet might facilitate the establishment of a microbiota optimized for efficient energy extraction and utilization from animal-derived nutrients.

### Progressive increase in microbial diversity during dietary transition

Alpha diversity analyses revealed a consistent increase in microbial richness and diversity from lecithotrophic to herbivorous and finally to carnivorous individuals (Table 1). Carnivorous individuals exhibited significantly higher richness and diversity than individuals at the other dietary stages. This pattern suggested that ontogenetic dietary transition was accompanied by associated microbiota expansion. A relatively low diversity observed in lecithotrophic individuals was biologically plausible because these individuals rely exclusively on endogenous yolk-derived nutrients and have limited exposure to exogenous microbes and dietary substrates. Studies of fish larvae have shown that microbial colonization begins early (including during yolk-sac stages), but the early microbiota is highly dynamic and strongly influenced by the initial microbial source and developmental stage, before stabilizing later in life (Fiedler et al., 2023). In addition, host development has been identified as a major driver of associated microbiota succession across larval-to-later stages in fishes (Xiao et al., 2021). The transition to herbivory might increase microbial diversity by introducing plant- or algae-associated and environmental microbes, while also broadening substrate heterogeneity through complex carbohydrates and polysaccharides. This interpretation was consistent with results of marine herbivorous fishes showing that associated microbes contributed to the breakdown and utilization of algal-derived compounds and that herbivorous fish harbored functional microbiota linked to digestion (Sparagon et al., 2022). Studies in European abalone likewise supported a relationship between digestive microbiota structure and algal diet composition (Gobet et al., 2018). Carnivory further increased alpha diversity in *H. tuba*, which might reflect the combined effects of prey-associated microbial inputs and the availability of protein- and lipid-rich substrates that support additional microbial guilds. More broadly, microbiome studies of the juvenile *Oncorhynchus keta* showed that the onset of feeding could drive major shifts and stabilization of associated microbiota, with diet acting as a strong source of microbial input (Ghosh et al., 2025). All the results showed that the observed increase in microbiota richness, diversity, and community turnover in *H. tuba* during the transition from lecithotrophy to herbivory and then to carnivory was driven by progressively greater microbial exposure and increasing dietary substrate complexity throughout ontogeny, a pattern consistent with previous studies in fish showing that associated bacterial colonization begins at the larval stage and that microbiota richness and diversity increase with yolk consumption and the onset of exogenous feeding, after which associated microbiota composition was closely associated with feeding habits across developmental stages (Wei et al., 2018).

Beta diversity analyses further confirmed that the carnivorous individuals harbored a significantly distinct microbial community (Figure 2). Both Bray–Curtis and UniFrac metrics consistently demonstrated a clear separation of carnivorous individuals from lecithotrophic to herbivorous individuals, indicating that the transition to a carnivorous diet represents a major turning point in microbial assembly. Comparable fish microbiome studies have also shown that diet-associated differences in community structure, supporting the interpretation that feeding strategy can restructure microbial assemblages (Liu et al., 2016).

However, several limitations of this study should be acknowledged. First, the sample size was relatively small, particularly in the He group, in which only four biological replicates met the requirements, which may have reduced the statistical power to detect subtle differences among dietary stages. Second, the functional profiles were inferred from 16S rRNA gene data using PICRUSt2 and were not experimentally validated; therefore, the enriched pathways should be interpreted as predicted functional potentials rather than direct evidence of metabolic activity. Third, although dietary transition was the primary factor considered in this study, host genotype effects could not be separated from dietary effects and may also have contributed to microbiota variation. Future studies should include larger sample sizes and integrate metagenomics, metatranscriptomics, metabolomics, and targeted biochemical validation to confirm the metabolic roles of the microbiota during dietary transition in *H. tuba*.

## Conclusion

This study demonstrated that the associated microbiota of *H. tuba* underwent stage-dependent restructuring across the transition from lecithotrophy to herbivory and carnivory, indicating that dietary shift was an important driver of microbial community assembly during early development. The identification of diet-associated microbial biomarkers further suggested that associated microbiota might play a role in nutritional adaptation during ontogenetic dietary transition. However, only four samples were retained in the He group after exclusion of one outlier sample, and the PICRUSt2-based functional predictions were not experimentally validated. Future research should validate these predicted functions using multi-omics and targeted experiments, and isolate key candidate indicator strains to assess their potential applications in *H. tuba* nutrition and aquaculture.

## Data Availability

The datasets presented in this study can be found in online repositories. The names of the repository/repositories and accession number(s) can be found in the article/Supplementary material.

## Supplementary material

SUPPLEMENTAL FIGURE 1 Rarefaction curves of 16S rRNA data and Shannon-Wiener diversity curves for 14 samples.

SUPPLEMENTAL FIGURE 2 The comparative analysis of the top 10 relative abundances of associated microbiota at the phylum, order, and genus levels, respectively, among the three dietary stage groups.
