# Supplementary figures and images for: The variation profile of associated microbiota in juvenile whelk Hemifusus tuba (Gmelin, 1791) in dietary transition
Source: Front Microbiol. 2026 May 28;17:1843060. doi: 10.3389/fmicb.2026.1843060 (PMC13267011; doi:10.3389/fmicb.2026.1843060)

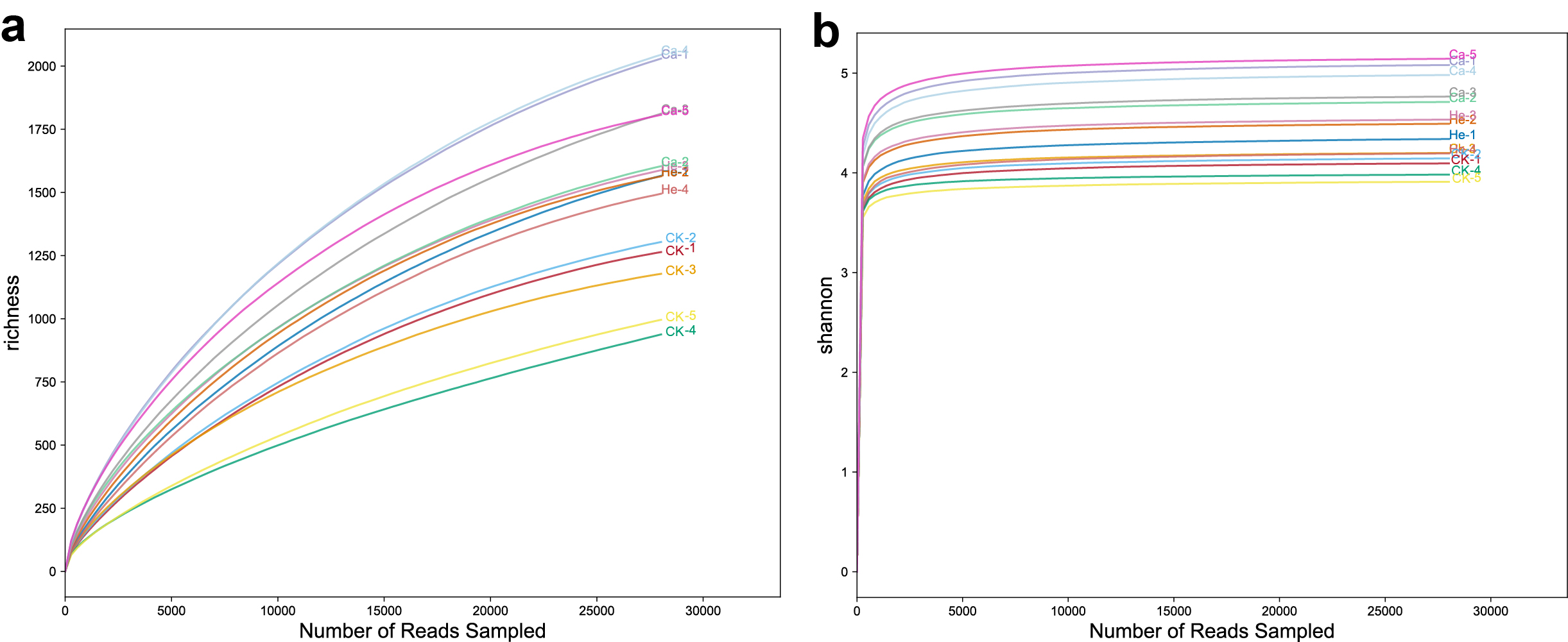

Supplement: SUPPLEMENTAL FIGURE 1 — Rarefaction curves of 16S rRNA data and Shannon-Wiener diversity curves for 14 samples. [file Image_1.jpeg]
